# Supplementary material for: Conditional chemoconnectomics (cCCTomics) as a strategy for efficient and conditional targeting of chemical transmission
Source: eLife. 2024 Apr 30;12:RP91927. doi: 10.7554/eLife.91927 (PMC11060718; doi:10.7554/eLife.91927)
Supplement: Figure 3—source data 3. [file elife-91927-fig3-data3.zip › Figure 3 source data3 part 2.zip!/Figure 3-Source data 3-part2/1053f2-1-CRISPResso_Report_y3hlioj1.zip!/CRISPResso_on_1053f2_2/4c.Quantification_window_insertion_deletion_substitution_locations.pdf]

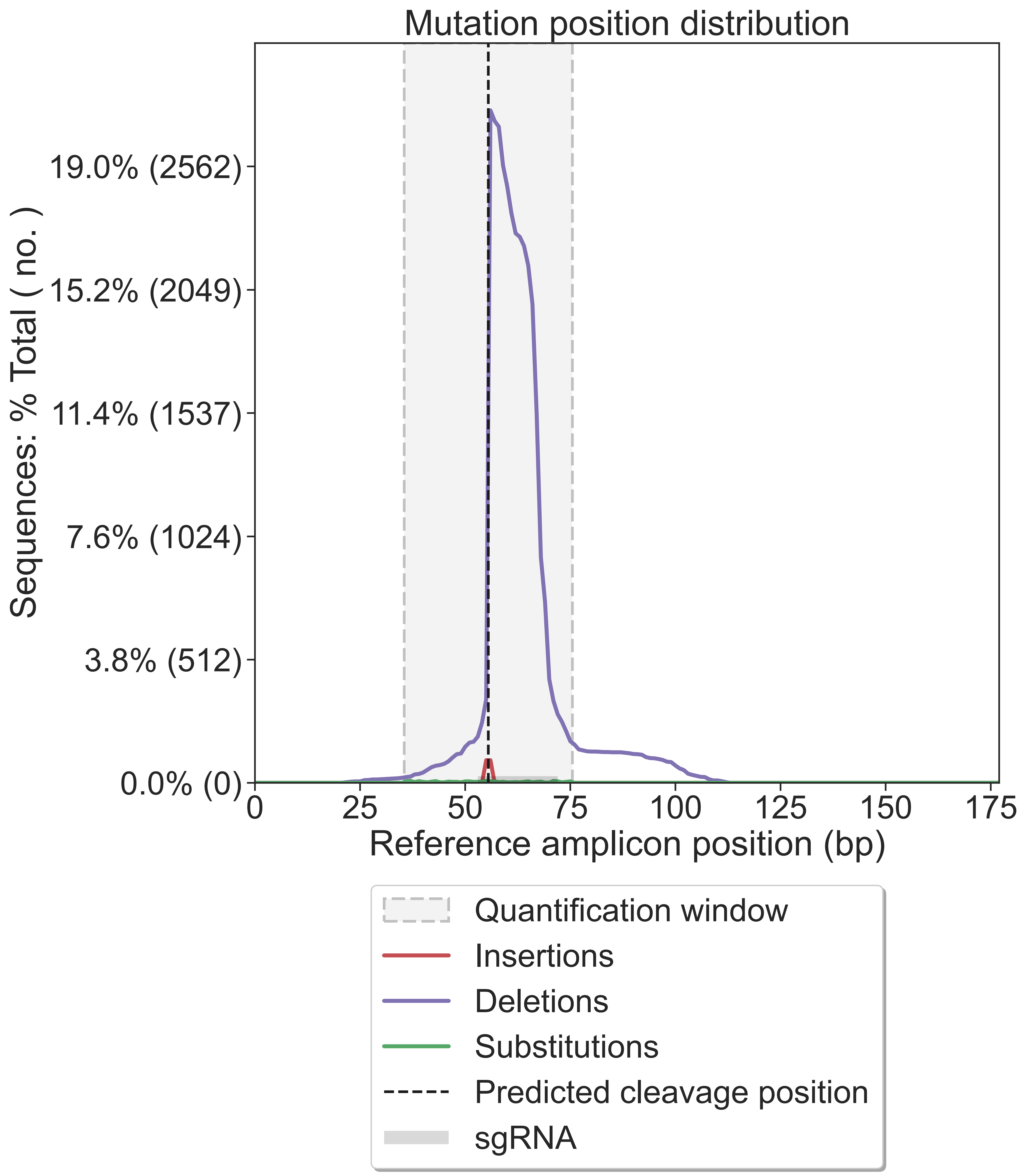
Mutation position distribution
Sequences: % Total (no.)
19.0% (2562)
15.2% (2049)
11.4% (1537)
7.6% (1024)
3.8% (512)
0.0% (0)
0
25
50
75
100
125
150
175
Reference amplicon position (bp)
Quantification window
Insertions
Deletions
Substitutions
Predicted cleavage position
sgRNA
